# Patients and Families’ Participation in Multidisciplinary Tumor Conferences Improves Patient and Family-Focused Cancer Care: Lessons Learned From a Debate on the Role of Radiation Therapy in Primary Mediastinal Non-Hodgkin Lymphoma

**DOI:** 10.7759/cureus.34693

**Published:** 2023-02-06

**Authors:** Hiba Z Ahmed, Mary R Nittala, Nivedha Kosalram, Betsy Crosswhite, Alice P Lee, Tracy C Frazier, Carter P Milner, Srinivasan Vijayakumar

**Affiliations:** 1 Radiation Oncology, University of Mississippi Medical Center, Jackson, USA; 2 Hematology and Medical Oncology, University of Mississippi Medical Center, Jackson, USA

**Keywords:** health literacy, multidisciplinary tumor conference, non-hodgkin’s lymphomas, deauville score, positron emission tomography computed tomography

## Abstract

Incorporation of patients’ preferences often leads to improved outcomes when included in the multidisciplinary tumor conference/board (MTC). However, patients’ wishes are not included or considered in the MTC decision-making. We need better strategies and approaches for patient-inclusive, shared decision-making. When finding ourselves at a crossroads regarding the next step in a patient’s treatment, we saw a unique opportunity for an MTC with the patient and her husband in attendance. The results of a full literature review regarding the role of consolidative radiation therapy (RT) in a patient with primary (thymic) B-cell lymphoma after completion of chemotherapy and fluorodeoxyglucose positron emission tomography-computed tomography (FDG-PET/CT) scan with a Deauville score of 4 were presented in a creative, engaging debate-style forum with visual aids. The patient and her husband were able to follow the discussion and, in the end, a consensus recommendation, heavily focused on the patient’s preferences, was offered and adopted, which ultimately resulted in the avoidance of excess treatment and likely improved her long-term quality of life outcome. These collaborative and innovative interactions benefit not only our patients but enrich our lives too as healthcare providers and strengthen us as a cancer care team in terms of understanding diversity in decision-making processes.

## Introduction and background

The use of multidisciplinary teams (MDTs) in patient care and treatment decision-making is not a recent development in the oncological healthcare setting [1]. MDTs help in improving team-based cancer care, as advances in individual specialties occur, rapid sharing of the expertise to improve outcomes becomes possible [2]. However, involving patients/families in *extensive *discussion during MDT meetings is a more recent phenomenon to address patients’ needs; secure coordination among professionals to prevent clinical errors; potentially improve outcomes such as survival, local control, and quality of life; and enhance patients’ social and psychological well-being for treatment and decision-making in difficult cases. One example of the use of an MDT is the multidisciplinary tumor conference/board (MTC), which enables healthcare providers to discuss the diagnosis and treatment methods for oncology patients and optimize patient-centered care. International studies have shown that decisions made in MTCs that incorporate patients’ treatment preferences yield better patient outcomes. However, MTCs often recommend treatment plans based on the analysis of data and results from clinical trials. From the healthcare provider’s standpoint, discussing treatment options with the patient and their families may be challenging due to patients’ lack of understanding of the intricacies of clinical cases. When professionals adjust their language and use simpler terms to communicate medical information with patients, possible misinterpretation of what the physician is trying to convey may increase a patient’s fear regarding his/her prognosis. This is more likely among patients/families with less education/health literacy (HL) [3-8]. In the state of Mississippi, the education level is unfortunately low among African Americans [9], who constitute about 40% of the population [10]. Further, the University of Mississippi Medical Center (UMMC) being a safety net hospital sees a higher percentage of African American patients.

To understand how patient involvement in MTCs yields benefits in patient outcomes, it is essential to know which patient subgroups participate in MTCs and evaluate their HL. A systematic review of 23 randomized trials of cancer patient decision-making summarized evidence on effective interventions to support shared decision-making [3]. A systemic review showed that the majority of patients preferred sharing decisions with physicians in 63% of the studies [4]. Table 1 depicts an overview of studies on patient participation and HL in MTCs.

**Table 1:** Summary of studies: patient characteristics and participation in MTCs. MTC = multidisciplinary tumor conference; n = number; % = percentage

Variables	Response	Study; n (%)			
		Heuser et al., 2019 [5]	Hahlweg et al., 2020 [6]	Whelan et al., 2003 [7]	Ravdin et al., 1998 [8]
Number of patients (n)		863	4,020	176	318
Participation in MTC	Yes	59 (6.8%)	2,749 (68.4%)	175 (99.4%)	98 (30.8%)
	No	804 (93.2%)	1,271 (31.6%)	1 (0.6%)	220 (69.2 %)
Education level	No school/No record	62 (7.2%)	681 (17.0%)	N/A	10 (3.2%)
	Lower secondary school	503 (58.3%)	969 (24.1%)	94 (53.7%)	28 (8.8%)
	Intermediate secondary school	101 (11.7%)	1,075 (26.7%)	81 (46.2%)	74 (23.2%)
	University entrance certificate	197 (22.8%)	1,295 (32.2%)	N/A	206 (64.8%)
Age (years)	Mean	51	58	51	49
	18–39	36 (4.2%)	N/A	18 (10.2%)	40 (12.6%)
	40–59	466 (54%)	N/A	102 (57%)	235 (73.9%)
	≥60	361 (41.8%)	N/A	55 (31.4%)	43 (13.5%)
Health literacy	Inadequate	139 (16.1%)	840 (20.9%)	28 (15.9%)	108 (34.0%)
	Problematic	287 (33.3%)	934 (23.2%)	92 (52.3%)	N/A
	Sufficient	437 (50.6%)	1,821 (45.3%)	51 (29.0%)	199(62.6%)
	Not reported	N/A	425 (10.6)	5 (2.8%)	11 (3.4%)
Tumor stage	Stage 0/I	441 (51.1%)	N/A	13 (7.4%)	146 (46.0%)
	Stage II	300 (34.8%)	N/A	75 (42.8%)	109 (34.3%)
	Stage III/IV	122 (14.1%)	N/A	87 (49.7%)	58 (18.2%)

In a prospective, multicenter, cohort study, a total of 863 newly diagnosed breast cancer patients were surveyed with 6.8% participation in MTCs and 61% sufficient HL among participating patients. Of the patients who did not participate in MTCs, sufficient HL scores were found in 49.9%, problematic HL in 33.3%, and inadequate HL in 16.8% of patients [5]. The analysis revealed that higher HL promoted better coordination among patients and their healthcare professionals and facilitated engagement in discussions regarding diagnosis and treatment. Conversely, patients with inadequate HL were less likely to participate in MTCs. Some studies reported higher rates of patient participation in MTCs (68.4% and 99.4%) with sufficient HL rates of 45.3% and 29.0% [6,7]. A survey of breast cancer patients concerning their knowledge and expectations of adjuvant therapy with 30.8% patient participation in MTC reported HL of 62.6%. A higher level of patient academic education is directly proportional to a higher rate of HL which is directly proportional to higher rates of patient participation in MTCs [8].

Another review study identified six key points of decision-making resulting from physician and patient-led discussions, namely, the definition of participation, importance of patient participation, factors influencing participation of patients in healthcare decisions, methods of patient participation, tools for evaluating participation, and benefits and consequences of patient participation in healthcare decision-making [11]. A systematic review of encounter patient decision aids showed a positive impact on patient-clinician collaboration. Despite facing implementation barriers, this study reported that the gaps in knowledge remain very effective and need to be prioritized [12].

Patients in the latter stages of the disease with incurable cancer chose treatment options recommended by their primary healthcare provider. For example, patients sought and adhered to a primarily physician-led decision when physicians assessed whether operative management was a viable modality of treatment or not. When asked why patients were encouraging these physician-led decisions, they stated that they lacked the time to travel and seek opinions from other specialists when their primary physician had already ruled out specific modalities of treatment, leading to greater acceptance of the primary physician’s choice of therapy. Patients also assumed that their diagnosis limited the treatment options available to them. In the case of likely incurable diseases, patients wanted the treatment which would give them the best chance of cure, and they often perceived that the best option, as suggested by the caregiver, was their only treatment choice [13]. In contrast, patients diagnosed with breast cancer in its earlier stages had a greater probability of discussing treatment options with their providers and making patient-led decisions. In these cases, patients were more likely to seek opinions from other breast cancer specialists to optimize their treatment decision-making. In some cases, patients even convinced caregivers to conduct additional tests to get a more accurate diagnosis. However, when patients were given two equivalent treatment options to choose from, they became distressed trying to decipher and assess the clinical information presented to them, again resulting in physician-led decisions. Patients reported that physician communication and the way information was presented to them acted as barriers to shared decision-making [14].

These studies overarchingly revealed that most treatment decisions are physician-led, and while some patients can voice their preferences in particular instances, even these patients find certain decisions distressing and rely on their physicians to make decisions on their behalf [11-14]. Studies such as the ones mentioned above illustrate the various nuances and clear need for a better understanding of our strategies and approaches to patient-inclusive decision-making [11-14].

## Review

Significance

Although MTCs are very common and have helped make team-based decisions in cancer care for many decades, involving patients/families as part of MTCs is less prevalent and varies from institution to institution with many geographical and national variations. In some countries, MDTs examine the patients together; review the images, pathology, and laboratory findings; and discuss the decisions in front of the patients or immediately in a conference setting before informing the patients/families. In other settings, MTCs occur not in a clinical setting, but rather in conference rooms. Nevertheless, a detailed debate in front of a patient or their family about the pros and cons of treatment options in an MTC is definitely not a common occurrence. This is the uniqueness of this report, with the understanding that such detailed MTCs may not be possible to make decisions for every patient. This review may help design such MTCs in the future under special circumstances on an as-needed basis. It has to be stated that such MTCs take time and effort to organize, manage, and coordinate, yet can be of great usefulness under certain circumstances.

Methodology

This paper does not fit into any typical format that most journals follow. The different way of presentation of this paper is due to the need to accommodate the *patient’s and her family’s voice* in the paper. We will first present the dilemma faced by the cancer care providers and in turn by the patient/family. Subsequently, the data will be presented in a debate format to recreate the way the MTC conducted its proceedings to serve as a model for future reference for others. In the end, we will discuss the importance of our approach as one of the ways to improve patient outcomes through patient/family participation in cancer care. No Institutional Review Board approval was required as this is not a research study; however, we obtained the patient’s consent to present the data and included the patient as a coauthor because she contributed to the intellectual content of the paper.

Case presentation

Ms. TCF, a 51-year-old female, was diagnosed with stage IB primary mediastinal (thymic) B-cell lymphoma (PMBCL) on 12/06/2021. Pathology showed the following molecular markers: CD117: negative, desmin: negative, LCA: positive within large cells of interest, mart 1: negative, pan-cytokeratin: positive within rare entrapped clusters of cells, vimentin: positive within large cells of interest, CD3: negative in large cells of interest, CD20: partially positive in large cells of interest, CD15: negative in cells of interest, CD30: partially positive in large atypical cells, PAX5: partially positive in large atypical cells. A fluorodeoxyglucose positron emission tomography-computed tomography (FDG-PET/CT) scan done on 01/12/2022 showed a 10 cm × 7 cm markedly hypermetabolic left upper mediastinal mass. She underwent a bone marrow biopsy on 01/14/22, which was negative for malignancy. She proceeded to undergo six cycles of dose-adjusted etoposide, prednisone, vincristine, cyclophosphamide, doxorubicin, and rituximab (DA-EPOCH-R) from 01/22/22 to 05/15/22.

An interim PET/CT done on 03/07/22 after two cycles of chemotherapy (specifically on day 20 of cycle two with the most recent chemotherapy on day five of cycle two, 02/30/22) showed significantly decreased hypermetabolism and size of the mass. The left paratracheal conglomerate, which initially had a maximum standardized uptake value (SUV_max_) of 2.7 and size of 5.0 × 4.6 cm, now had an SUV_max_ of 1.3 and measured 5.4 × 4.7 cm. The left anterior mediastinal mass, which previously had an SUV_max_ of 18.6 and measured 9.5 × 9.2 cm now had a SUV_max_ 3.3 and measured 5.6 × 4.9 cm. A Deauville score of 4 was assigned.

An end-of-treatment (EOT) PET/CT done on 06/01/22 after six cycles of chemotherapy (specifically on day 22 of cycle six with the last chemotherapy on day five of cycle six, 05/15/22) showed a continued favorable response. The left paratracheal conglomerate now had an SUV_max_ of 2.0 and measured 4.6 × 3.7 cm (previous SUV_max_ 2.0 and size of 5.2 × 3.9 cm by the new radiologist reading the scan), resulting in a Deauville score of 4. The left anterior mediastinal mass now had an SUV_max_ of 1.5 and a size of 4.2 × 3.1 cm (previous SUV_max_ of 2.0 and size of 5.2 × 3.3 cm by the new radiologist reading the scan), resulting in a Deauville score of 3. With an EOT PET/CT showing a Deauville score of 3 for one focus of disease and a Deauville score of 4 for the other focus of disease, Ms. TCF was referred to radiation oncology for consolidative radiation therapy (RT). With a Deauville score of 4, should Ms. TCF receive further therapy?

Format of the multidisciplinary tumor conference/board

A debate format was employed to research and present data. A full literature review was conducted by each side of the debate for versus against consolidative RT. Each party used data published from clinical trials conducted since 2013 focusing on the most recent findings for the patient’s specific tumor type, PMBCL, and the treatment option under investigation, consolidative RT. They compiled highlights from various articles into a visual presentation that was used as supporting evidence at the MDT gathering.

Careful consideration was given to securing an MDT including disciplines and personnel invited. The Chair of the Department of Radiation Oncology at UMMC led the MDT and the argument in favor of consolidative RT. The opposing argument was conducted by the Chief Resident of the Department of Radiation Oncology at UMMC. The patient’s hematologist-oncologist was also invited to play a leadership role in the debate. Attendees included radiation oncology faculty, staff, and residents, as well as hematology-oncology faculty, residents, and the patient and her husband.

The debate began with the Chair of Radiation Oncology at UMMC introducing the topic of discussion and setting the scene for the conference. Participants gathered in person as well as online via Webex. For the first 15 minutes, the affirmative representative argued for treatment with RT in non-Hodgkin lymphoma (NHL). He used publications from the previous full literature review to support his argument. The opposition then presented a case against RT specifically for PMBCL. Her argument was divided into three points, each with supporting publications. Following the initially proposed methods of treatment, each party was offered a five-minute rebuttal. The affirmative party continued to support the use of RT while the opposition used her five-minute rebuttal to argue against this treatment modality. This concluded the prepared presentations. At this time, the hematology-oncology faculty was provided time to comment on the proposed treatment plans and the evidence presented. Then, the radiation oncology faculty was allotted additional comments. After the discussion, the patient’s hematologist-oncologist offered concluding remarks and her opinion on the treatment plan. The debate was concluded by the patient and her husband speaking to the MDT. They shared their thoughts and ultimately decided which treatment plan they would like to pursue.

It was of the utmost importance to include the patient and her husband in the debate. Key to a multidisciplinary approach is patient involvement in the treatment planning process. The patient’s presence at the debate enabled her to make a fully informed decision regarding her personalized cancer care.

Details of the proceedings of the multidisciplinary tumor conference/board

Data for Radiation Therapy

Diffuse large B-cell lymphoma (DLBCL) is a biologically heterogeneous disease even within the early stages [15,16]. Hence, there is a need to individualize each patient’s treatment. Before the use of chemotherapy, RT was the standard therapy for DLBCL. With the advent of chemotherapy alone or RT plus chemotherapy, the outcomes have improved. The South West Oncology Group (SWOG) randomized study (SWOG 8636) was a non-inferiority phase III clinical trial comparing eight cycles of cyclophosphamide, doxorubicin, vincristine, and prednisone (CHOP) versus CHOP plus RT. Chemotherapy alone versus three cycles of CHOP plus RT (CHOP3RT) led to a more judicious use of consolidative RT [17]. Although initial five-year results showed better progression-free survival (PFS) and overall survival (OS) for CHOP3RT, long-term results showed no significant differences. In the pre-rituximab (an anti-CD-20 monoclonal antibody) era, using stage-modified international prognostic index (smIPI) in the limited-stage disease, the OS with no risk factors was 95% versus 75% with one to two risk factors; this worsened to 50% with three or more risk factors. These outcomes in terms of OS improved in the rituximab era [15].

**Figure 1:**
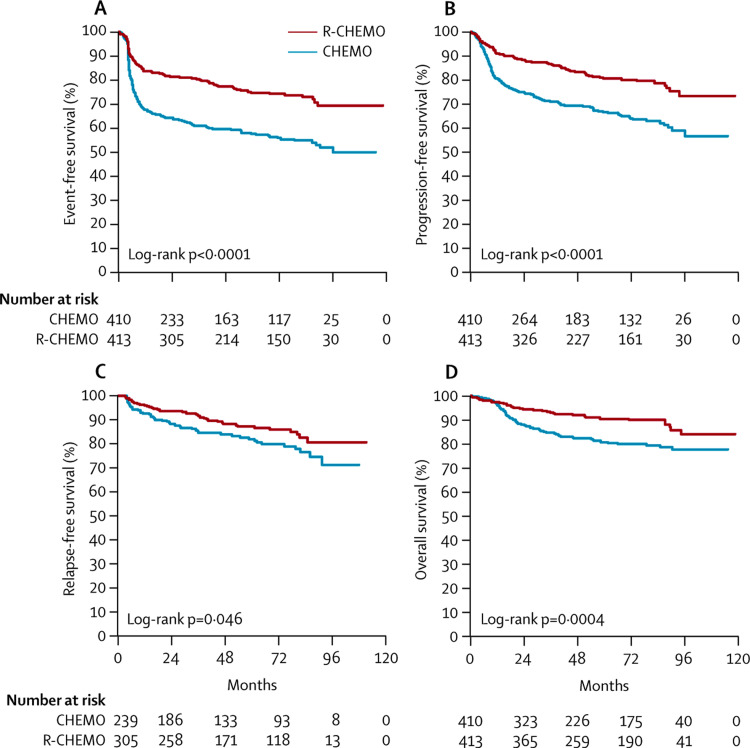
Event-free (A), progression-free (B), relapse-free (C), and overall (D) survival of 823 patients assigned to six cycles of CHOP-like chemotherapy or the same chemotherapy plus six applications of rituximab. CHEMO = chemotherapy; R-CHEMO = chemotherapy plus rituximab This image is reproduced from Pfreundschuh et al. [18]. Permission to reproduce was obtained from the licensed content publisher Elsevier.

Immunochemotherapy (IchT) using rituximab plus chemotherapy (usually CHOP) further improved the outcomes in NHL [18]. The MabThera International Trial (MinT) comparing CHOP-like chemotherapy versus IchT with the addition of rituximab in young patients with limited-stage DLBCL (with <1 age-adjusted international prognostic index (IPI) and age group of 18-60 years) showed improved six-year outcomes in the IchT arm. Although the IchT arm had better outcomes, this was even more dramatic if there was bulky disease and/or IPI of 1, a fact that is important for our patient. For example, the event-free survival (EFS) at six years was 84% for those with non-bulky disease and/or IPI of 0 versus 71% (p = 0.005) for those with bulky disease and IPI of ±1. These results argued in favor of consolidative RT for our patient (Figure 1).

However, the finer details reported in the web appendix of the cited paper showed that the bulky disease might have been treated with RT in a subset of patients as per the choices of the treating physicians to a dose of 30-40 Gy. These data lead us to believe there needs to be other supporting evidence to strongly recommend radiotherapy (see below) [19,20].

However, the use of RT for bulky disease can be recommended based on two other studies. The MD Anderson Cancer Center retrospectively analyzed the use of involved-field RT (IFRT) after six cycles of R-CHOP [19]. The PFS was 92% versus 73% at five years. There were no disease failures within the IFRT fields. Similar conclusions can be drawn from a more recent study [20]. This is another retrospective study from the pre-PET era. Another shortcoming of this paper in terms of applying the results to make decisions for our patients is that the study was confined to stages III and IV patients. Nonetheless, the benefits of consolidative RT were remarkable: (a) the median OS for the chemotherapy-only arm was six years; for the chemotherapy + RT cohort, the median OS had not been reached yet; (b) the benefit from adding RT was an additional 36% (85.9% vs. 49.2%, p = 0.0001); (c) absolute PFS benefit was 14.5% (87.4% vs. 72.9%, p = 0.013]; (d) 37% absolute benefit in local control rates was noted (91.9% vs. 54.9%, p = 0.0001).

**Figure 2:**
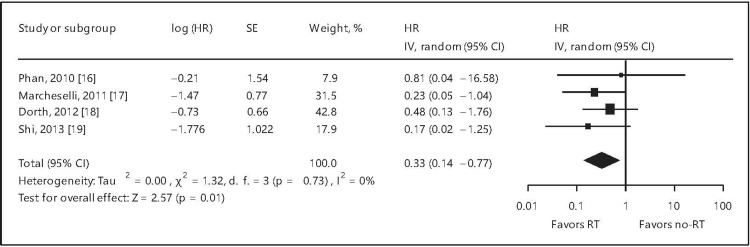
Forest plot of overall survival for all patients. HR = hazard ratio; SE = standard error; CI = confidence interval; RT = radiation therapy; % = percentage This image is reproduced from Hu et al. [21]. Permission to reproduce was obtained from the licensed content publisher Karger.

**Figure 3:**
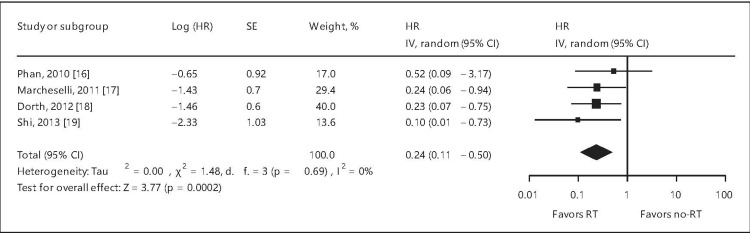
Forest plot of progression-free survival/event-free survival for all patients. HR = hazard ratio; SE = standard error; CI = confidence interval; RT = radiation therapy; % = percentage This image is reproduced from Hu et al. [21]. Permission to reproduce was obtained from the licensed content publisher Karger.

**Figure 4:**
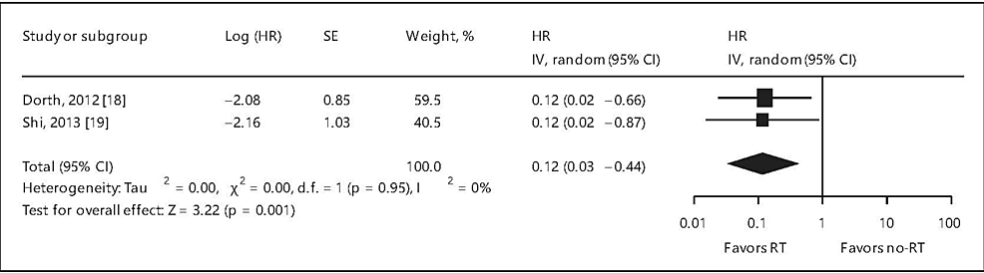
Forest plot of local control for patients with stage III-IV diffuse large B-cell lymphoma. HR = hazard ratio; SE = standard error; CI = confidence interval; RT = radiation therapy; % = percentage This image is reproduced from Hu et al. [21]. Permission to reproduce was obtained from the licensed content publisher Karger.

A meta-analysis unequivocally affirmed the role of RT in DLBCL. The forest plots for OS, PFS, and local control (LC) are depicted in Figures 2-4 and confirm the advantages of RT in DLBCL [21].

An editorial pointed out the need to address the following in the subsequent clinical trials: (a) the role of PET/CT in RT decision-making; (b) the incorporation of interim PET-CT response in RT considerations; (c) IFRT versus involved-node RT (INFT); (d) the dose of RT (30 Gy versus more); (e) the role of RT in salvage and refractory to IchT setting; and (f) the role of tissue and blood biomarkers in RT recommendations [21,22].

**Figure 5:**
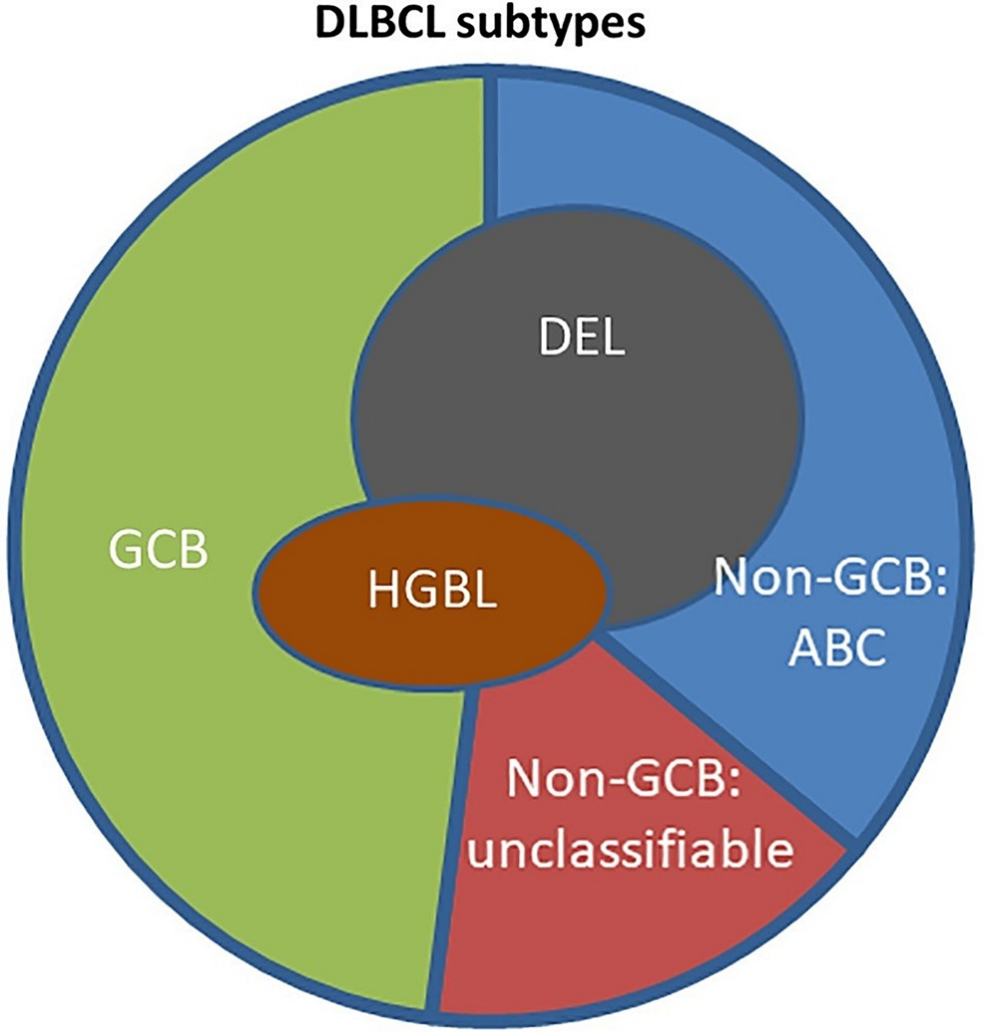
Overlap of DLBCL subtypes by COO and molecular features. DLBCL = diffuse large B-cell lymphoma; COO = cell of origin; GCB = germinal center B-cell-like; ABC = activated B-cell-like; HGBL = with MYC and BCL2 and/or BC16, also known as double/triple HIT; DEL = double expresser lymphoma; MYC and BCL2 and/or BC16 = oncogenes commonly deregulated in lymphomas This image is reproduced from Liu et al. [16]. Permission to reproduce was obtained from the licensed content publisher John Wiley and Sons.

In the considerations of RT, the modern view of DLBCL need to be considered [16]. The current concepts on the determination of outcomes include clinical features, cell of origin (COO) of the NHL, molecular features, and recurring mutations (Figure 5).

The role of RT based on DLBCL molecular subtypes is still evolving. For example, should RT be standard in the activated B-cell-like (ABC) subtype? ABC DLBCL carries a poorer outcome than the germinal center B-cell-like (GCB) subtype, with five-year OS rates of 45% versus 80%. While the decisions on the types of upfront systemic therapies based on molecular subtypes are being debated, the role of RT based on similar considerations still needs to evolve. Given the above, how do we recommend further treatments for our patient? We need to follow the path of the Risk-Adapted Treatment Decision-Making Process (RATDMP).

**Figure 6:**
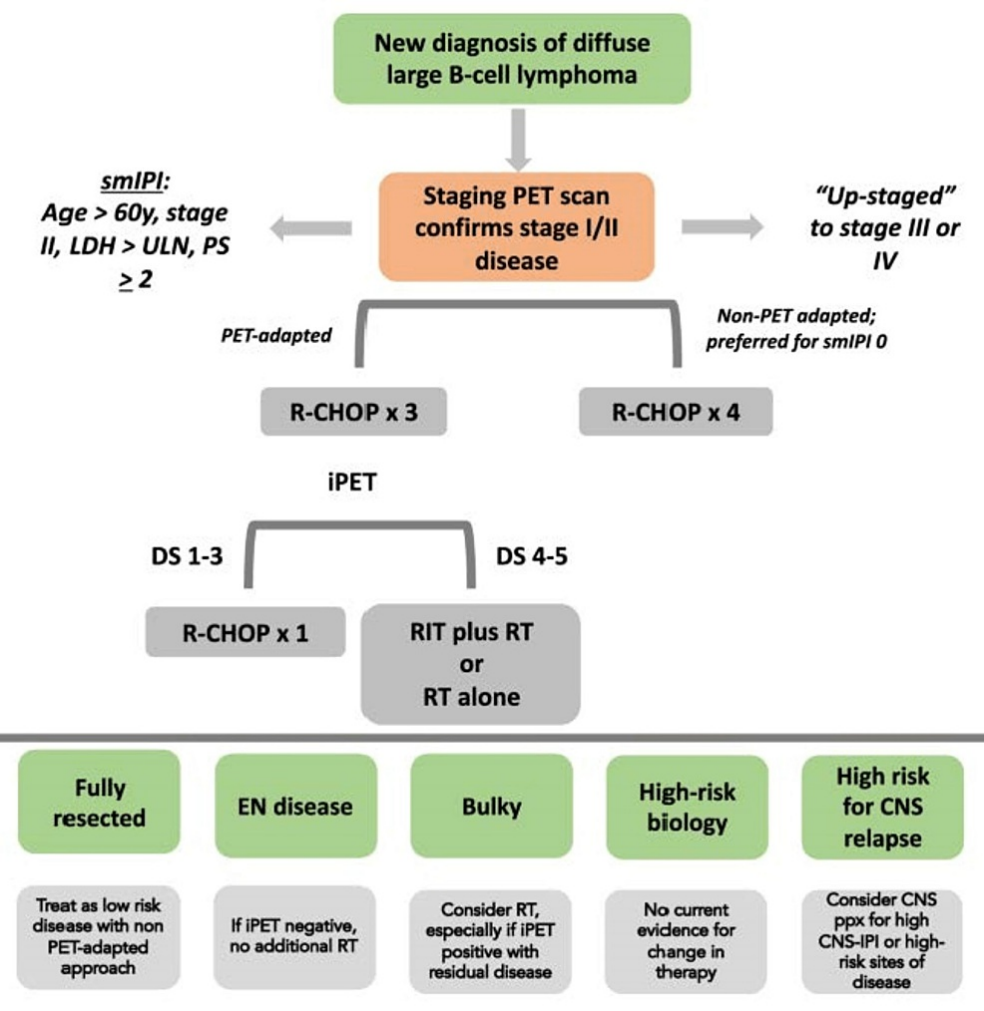
Approach to the management of limited-stage diffuse large B-cell lymphoma. iPET = interim positron emission tomography; smIPI = stage-modified international prognostic index; RT = radiation therapy; RIT = radioimmunotherapy; EN = extranodal; DS = Deauville score; ppx = prophylaxis; PS = performance status; ULN = upper limit normal; CNS = central nervous system This image is reproduced from Rojek et al. [15]. The image is available via Creative Commons Attribution 4.0 International License.

Figure 6 outlines the RATDMP [15]. The increasing role of PET-CT (at the time of initial diagnosis and staging as well as part of interim response evaluation), the bulkiness of the disease, and COO considerations is obvious from Figure 6 in the circa 2020s.

In the further discussion, the focus will be limited to stage DLBCL with bulky initial extent disease, as was needed to help our patient. For this, the following have to be recognized: (a) in stage I, the bulky disease is a rare entity; (b) the definition of the bulky disease varies from study to study; (c) most clinical trials (especially those including stage I disease) unfortunately exclude bulky disease patients (e.g., FLYER, LYSA/GOELAMS 02-02, SWOG 0014, 21001). These facts lead to making RT decisions in stage I bulky disease in the PET-CT era very difficult, involve the application of the art of medicine, and are not based on the science of medicine. This approach is even more essential in PMBCL in stage I bulky disease. In this respect, the British Society of Hematology (BSH) guidelines are very helpful when limited evidence exists based on science alone. BSH used the Grading of Recommendations Assessment, Development, and Evaluation (GRADE) nomenclature. Furthermore, these guidelines shown in Table 2 and Table 3 were developed in 2019 (in the modern PET-CT era) specifically for PMBCL [23].

**Table 2:** The GRADE nomenclature used to evaluate the quality of evidence and assess the strength of recommendations. GRADE = The Grading of Recommendations Assessment, Development, and Evaluation This table is reproduced from Cwynarski et al. [23]. Permission to reproduce was obtained from the licensed content publisher John Wiley and Sons.

GRADE nomenclature
Strong (grade 1): Strong recommendations (grade 1) are made when there is confidence that the benefits do or do not outweigh harm and burden. Grade 1 recommendations can be applied uniformly to most patients. Regard as “recommend”
Weak (grade 2): Where the magnitude of benefit or not is less certain a weaker grade 2 recommendation is made. Grade 2 recommendations require judicious application to individual patients. Regard as “suggest”
Quality of evidence
The quality of evidence is graded as high (A), moderate (B), or low (C). To put this in context, it is useful to consider the uncertainty of knowledge and whether further research could change what we know or our certainty
(A) High: Further research is very unlikely to change confidence in the estimate of effect. Current evidence derived from randomized clinical trials without important limitations
(B) Moderate: Further research may well have an important impact on confidence in the estimate of effect and change the estimate. Current evidence derived from randomized clinical trials with important limitations. (e.g., inconsistent results, imprecision wide confidence intervals, or methodological flaws such as lack of blinding, large losses to follow up, and failure to adhere to intention to treat analysis), or very strong evidence from observational studies or case series (e.g., large or very large and consistent estimates of the magnitude of a treatment effect or demonstration of a dose-response gradient)
(C) Low: Further research is likely to have an important impact on confidence in the estimate of effect and is likely to change the estimate. Current evidence from observational studies, case series, or just opinion

**Table 3:** British Society of Hematology recommendations. This table is reproduced from Cwynarski et al. [23]. Permission to reproduce was obtained from the licensed content publisher John Wiley and Sons.

Immunochemotherapy recommendations
• Patients should be offered a clinical trial wherever possible (1A)
• R-CHOP (rituximab, cyclophosphamide, doxorubicin, vincristine, and prednisolone) × 6+ involved-site radiotherapy (ISRT) is standard of care for treatment of PMBCL (1B)
• DA-EPOCH-R (dose-adjusted etoposide, prednisone, vincristine, cyclophosphamide, doxorubicin and rituximab) × 6 without ISRT is an alternative approach but should only be given in centers experienced in the delivery of complex chemotherapy regimens (1B)

Given the lack of randomized studies in stage I bulky PMBCL, we have to use the art of medicine in decision-making and IFRT is recommended.

Data Against Radiation Therapy

Our patient had PMBCL and not another form of NHL. It is histologically, biologically, and clinically more closely related to nodular sclerosing Hodgkin lymphoma (nsHL) than an NHL. Histologically, PMBCL overlaps with nsHL in that PMBCL expresses B-cell markers (such as CD19, CD20, CD22, and CD79a) but lacks expression of surface or even cytoplasmic immunoglobulin (Ig), despite expression of the Ig co-receptors/antigens (such as CD79a), unlike other B-cell lymphomas [24]. Biologically, it is also similar to nsHL in that there is constitutive activation of the Janus kinase (JAK) signal transducer and activator of transcription (STAT) and nuclear factor kappa light-chain enhancer of activated B cells (NF-κB) pathways [25]. Clinically, similar to nsHL, it primarily presents as a bulky mediastinal mass in adolescents and young adults and is more common in females (unlike other NHL subtypes).

**Figure 7:**
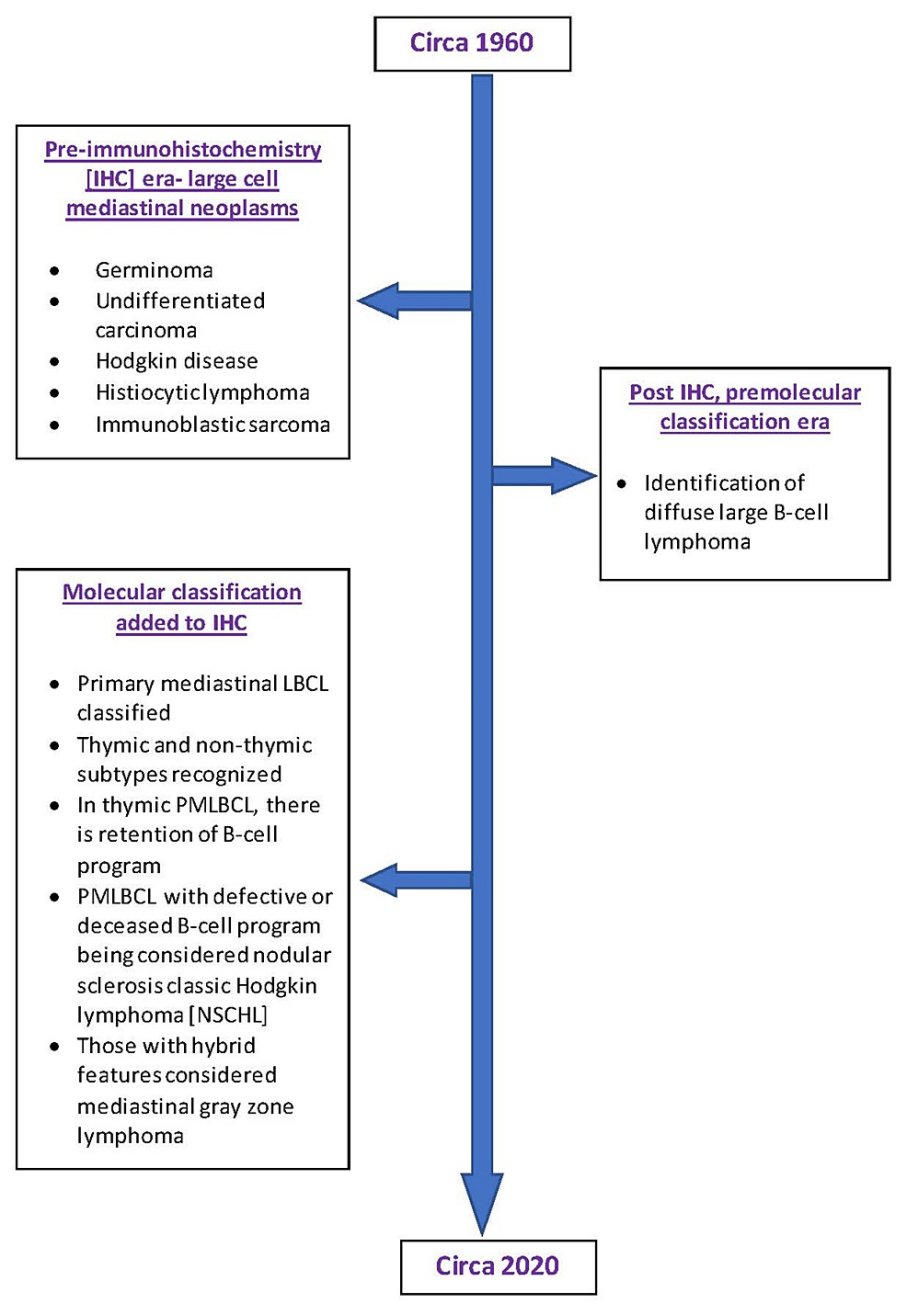
Evolution of morphology and immunohistochemistry. IHC = immunohistochemistry; LBCL = large B-cell lymphoma; PMLBCL = primary mediastinal large B-cell lymphoma; NSCHL = nodular sclerosis classic Hodgkin lymphoma This image is a simplified depiction from the authors of this paper.

From a historical perspective, large-cell lymphoma is the anterior mediastinum that has evolved over the last seven decades. Figure 7 depicts this evolution of morphology and immunohistochemistry as we have come to better understand this entity.

Through the evolution of morphology and immunohistochemistry, we have come to better understand this entity. In 2003, PMBCL became better understood, and through gene profiling and cytogenetics, it can be distinguished from DLBCL-not otherwise specified [26].

**Figure 8:**
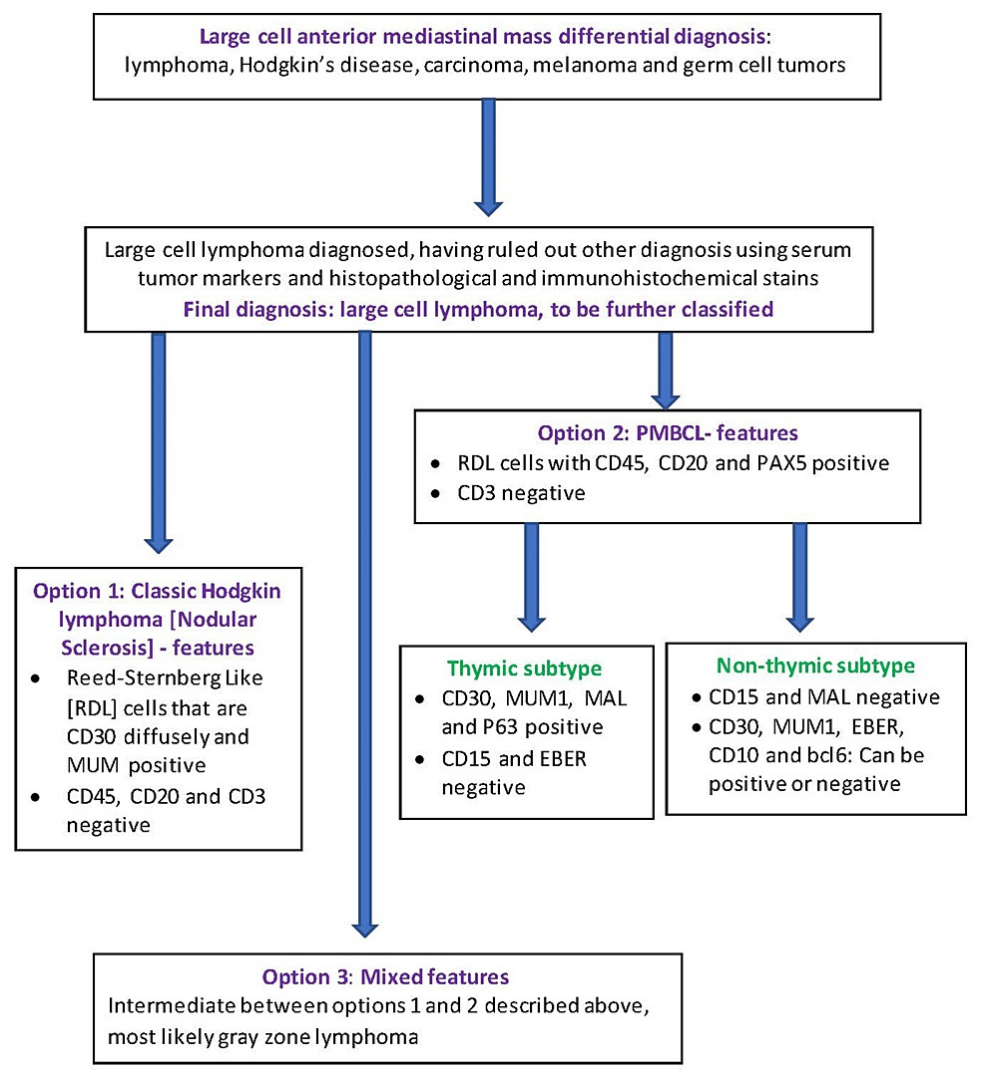
Pathologically diagnosing an anterior mediastinal large-cell neoplasm, with a focus on primary mediastinal large B-cell lymphoma. PMLBCL = primary mediastinal large B-cell lymphoma; RDL = Reed-Sternberg like; EBER = Epstein-Barr encoding region; CD45, CD30, CD20, CD15, CD10, CD3, PAX5, P63, MUM1, MAL, bc16 = markers This image is a simplified depiction from the authors of this paper.

Typically diagnosed from a core needle biopsy of a patient’s anterior chest mass, PMBCL exhibits large cells and Hodgkin/Reed-Sternberg-like cells that are CD45+, CD20+, PAX5+ strong, and C3-. The differential diagnosis of a primary mediastinal core biopsy with large cells includes classical Hodgkin lymphoma, PMCBL including non-thymic subtype, and mediastinal gray zone lymphoma. The spectrum of these lymphomas is closely connected, and it is important to recognize the immunohistochemical differences between these lymphomas which guide our pathologists to a correct diagnosis (Figure 8). Thymic PMBCL are further characterized by findings including heterogeneous CD30+, MUM1+, CD15-, and EBER-, which can be used to distinguish from non-thymic PMBCL [27].

In 2003, PMBCL became better understood, and through gene profiling and cytogenetics, it can be distinguished from DLBCL-not otherwise specified.

Our patient was treated with DA-EPOCH-R, not other chemotherapy regimens. In a New England Journal of Medicine prospective phase II study, 51 patients with untreated PMBCL were treated with DA-EPOCH-R. The primary objectives of the study were the rate of complete response (CR), PFS, and the toxicity of DA-EPOCH-R. Patients who had a standard uptake value (SUV) > mediastinal blood pool uptake in any residual mass underwent repeat PET-CTs until normalization or stabilization. A tumor re-biopsy was performed as clinically indicated. None of these patients received any further treatment unless they had a biopsy-proven refractory or relapsed disease. For the 51 enrolled patients, the median age was 30 years (19-52), the median tumor diameter was 11 cm, and 59% of the patients were women. At a median follow-up of 63 months, EFS was 93%, and OS was 97%. The use of DA-EPOCH-R obviated the need for RT in all but two of 51 (4%) patients [28].

A single EOT PET-CT has poor sensitivity. According to the Journal of Clinical Oncology published consensus guidelines by International Conference on malignant lymphomas imaging working group [29], nonspecific FDG uptake may occur with treatment-related inflammation. Patients should be scanned as long after the previous chemotherapy administration as possible for the interim assessment. A minimum of three weeks, but preferably six to eight weeks, after completion of the last chemotherapy cycle, two weeks after granulocyte colony-stimulating factor (GCSF) treatment, or three months after RT is recommended. As a reminder, our patient’s EOT PET-CT (6/1/22) was done only 2.5 weeks after her last chemotherapy (5/15/22). As a continuation of the previous prospective phase II study in which patients diagnosed with untreated PMBCL were treated with DA-EPOCH-R, 93 patients (59 from the previously published prospective National Cancer Institute (NCI) and 34 from retrospective Stanford data with eight new NCI and 18 new Stanford patients) were included in a study evaluating serial imaging with PET-CTs [30]. Overall, 80/93 patients had EOT PET/CTs. Moreover, 35/55 patients with negative EOT PET-CT (Deauville score 1-3) underwent serial PET-CTs. Finally, 22/25 patients with positive EOT PET/CT (Deauville score 4-5) underwent serial PET-CTs. With a median follow-up of 8.4 years, EFS at eight years was 90.6%, and OS at eight years was 94.7%. EOT PET/CT had a positive predictive value (PPV) of 20%, but a negative predictive value (NPV) of 98%. In the six treatment failures that occurred, the median EOT PET-CT SUV_max_ was 15.4 (1.9-21.3). Overall, 2% of patients with an EOT PET-CT Deauville score of 1-3 recurred, 6% of patients with an EOT Deauville score of 4 recurred, and 50% of patients with an EOT Deauville score of 5 recurred (Table 4).

**Table 4:** EOT PET-CT response following DA-EPOCH-R therapy. EOT = end of treatment; PET-CT = positron emission tomography-computed tomography; n = number; % = percentage This table is reproduced from Melani et al. [30]. This image is available via Creative Commons License CC BY-NC through the Ferrata Storti Foundation.

Lymphoma status	Deauville score				
(N = 80 total with EOT FDG-PET)					
	Negative (55/80, 69%)			Positive (25/80, 31%)	
	1	2	3	4	5
	30%	24%	15%	21%	10%
No treatment failure - patients (n)	24	18	12	16	4
Treatment failure - patients (n)	0	1	0	1	4

Patients with negative EOT PET-CT rarely need and are unlikely to benefit from additional RT (98% never progressed and 2% progressed). Most patients with a positive EOT PET-CT achieve long-term remission and would not benefit from empirical consolidative RT. Imaging with serial PET-CTs is a highly effective strategy to distinguish the persistent disease from post-treatment inflammatory changes. Overall, the use of serial PET-CTs reduced the percentage of patients who would receive RT from 31% (if empirically treated) to 5% (biopsy-confirmed treatment failure).

The unique biology of PMBCL results in a high false-positive rate. There needs to be a paradigm shift in clinical decision-making for patients who receive DA-EPOCH-R. Singular EOT PET-CT does not accurately identify treatment failures. Serial imaging with PET-CTs effectively discriminates between patients with true residual disease versus post-treatment inflammatory changes. Serial PET-CTs should be done for patients with an initial positive EOT PET-CT to identify treatment failures that require RT.

Rebuttal findings

Rebuttal for Radiation Therapy

**Figure 9:**
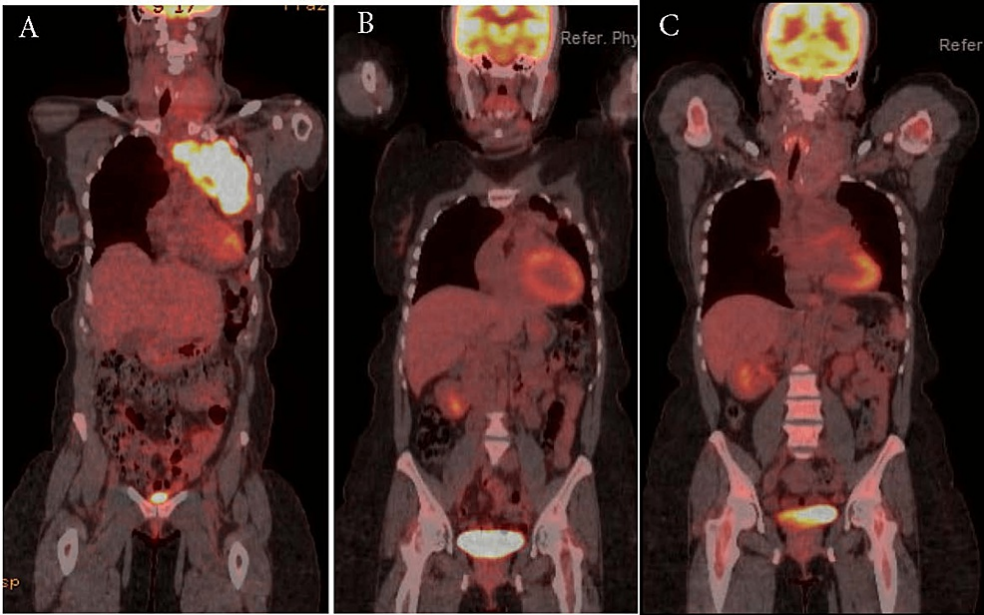
PET-CT of the UMMC patient before and during the IchT. PET-CT = positron emission tomography-computed tomography; UMMC = University of Mississippi Medical Center; IchT = immunochemotherapy

Our patient was treated with six cycles of DA-EPOH-R and yet had a Deauville score of 4 after the completion of IchT (Figure 9).

Let us look at what the Deauville score of 4 means (Table 5) [31]. The fact that the PET-CT identified mediastinal mass cephalad to the heart makes the risk-benefit ratio in favor of IFRT because the cardiac dose constraints can be easily met with intensity-modulated RT (IMRT) and image-guided RT (IGRT).

**Table 5:** The Deauville five-point scoring system. Scores of 1 and 2 are considered to be negative, Scores of 4 and 5 are considered to be positive, and Score 3 is interpreted according to the clinical context. This table is adapted from Follows et al. [31]. Permission to reproduce was obtained from the licensed content publisher John Wiley and Sons.

Scoring	Description
Score1	No uptake above the background
Score 2	Uptake ≤ mediastinum
Score 3	Uptake > mediastinum but ≤ liver
Score 4	Uptake moderately increased compared to the liver at any site
Score 5	Uptake markedly increased compared to the liver at any site
Score X	New areas of uptake unlikely to be related to lymphoma

**Figure 10:**
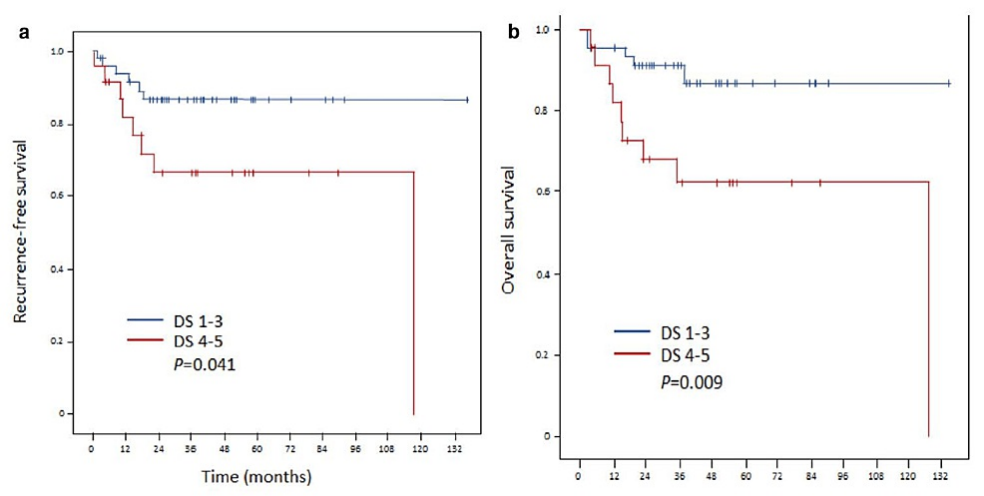
The prognostic value of PET-CT evaluation with DS on the recurrence and survival in diffuse large B-cell lymphoma: a multi-institutional study of KROG 17-02. (a) Locoregional recurrence-free survival and distant failure-free survival rates after propensity score matching (1:2) for the DS 1-3 and 4-5 arms. (b) Recurrence-free survival and overall survival rates after propensity score matching (1:2) for the DS 1-3 and 4-5 arms. KROG = The Korean Radiation Oncology Group; DS = Deauville score This image is reproduced from Lee et al. [32]. Permission to reproduce was obtained from the licensed content publisher Springer Nature.

A recent study evaluating the Deauville scores on the recurrence and survival of DLBCL analyzed the data from clinical trials conducted by the Korean Radiation Oncology Group (KROG) in terms of OS, PFS, and LC rates between post-IchT PET-CT results. This study compared outcomes between Deauville scores of 1-3 and 4-5. Twenty-four patients were identified with Deauville scores of 4-5; those with Deauville scores of 1-3 were matched by a 1:2 ratio (48 patients). With a median follow-up of 37 months, PFS was 86% versus 66% (p = 0.041), and OS of 87% versus 62% (p = 0.009). These differences were sustained in the multivariate analysis also with a hazard ratio of 3.8 for PFS and 4.5 for OS in favor of those with Deauville scores of 1-3 versus Deauville scores of 4-5. The PFS and OS curves comparing the two groups are shown in Figure 10 [32].

Despite the striking results of KROG analysis, the UMMC radiation oncologist leaned toward a wait-and-repeat PET-CT recommendation for the UMMC patient for the following reasons: (a) KROG data were based on a small number of patients; (b) it was not designed to prospectively study the question; and (c) the timing of our patient’s post-IchT evaluation PET-CT might have been too early. Given the above, the recommendations by the lead UMMC radiation oncologist are indicated in Table 6.

**Table 6:** Recommendations by the UMMC lead radiation oncologist. PET-CT = positron emission tomography-computed tomography; RT = radiation therapy; PFT = pulmonary function test; IMRT = intensity-modulated radiation therapy; IGRT = image-guided radiation therapy; 4D = CT done taking into consideration the tumor with breathing motion; Gy = gray; MRI = magnetic resonance imaging

Wait and repeat PET-CT
• Do “no harm”
• Repeat PET-CT in a month
• If PET-CT is still positive, treat with RT to the involved site
• If the patient wants treatment now, treat with RT to the involved site
• Listen to our patients and respect their informed consent decisions
If radiotherapy is given:
• Avoid the heart completely
• Get an Echo and determine cardiac ejection fraction
• Get PFT
• RT: use IMRT and IGRT techniques
• 4D-based simulation
• Image Fuse with the first PET-CT; yet, cut off the lung aspect of the mass protruding into the lung
• Only to the upper mediastinum
• Decrease the lung dose as much as possible
• Total dose: 30 Gy
• Fraction size: 2 Gy
• Follow-up: PET-CT, mammograms, periodic breast MRIs

Rebuttal Against Radiation Therapy

The data presented by the opposition in favor of consolidative RT primarily pertains to DLBCL when PMBCL is its entity and is far more closely related to NHL histologically, biologically, and clinically than DLBCL. The data presented by the opposition in favor of consolidative RT primarily refers to data on older chemotherapy regimens, such as R-CHOP, which are not the newest accepted standard-of-care chemotherapy regimen for PMBCL, which is DA-EPOCH-R. The PET-CT data used by the opposition referred to the results of the interim PET-CT and not an EOT PET-CT or serial PET-CTs after completion of chemotherapy, which studies have proven should be given greater consideration than the interim PET-CT when determining the role of consolidative RT for PMBCL.

The patient and her husband’s comments

NHL, the word itself is frightening. I was simply going to get an MRI of my breast and there it was. I asked my doctor if he was joking and he said, no. This was the beginning of my journey. The second step was to get a CT with contrast of the area. My doctor then referred me to a medical oncologist. During the meeting with the medical oncologist, she told me that she needed more information about lymphoma. She ordered a bone aspiration and a CT biopsy of the affected area. The results revealed little about the bulky mass. She shared information about the sum of five days of chemotherapy per setting with four to six settings. I stopped hearing her after this information. My husband gently touched me, and I began to listen again. She mentioned something about EPOCH-R and needing to see a hematologist instead of a medical oncologist.

Meeting my hematologist was a much-needed rainbow after my storm. She was kind and comforting. I listened as she explained my bulky mass. She spoke with great confidence about my prognosis. She gave me choices that I had not heard of before. After the initial meeting, I decided that I was ready for treatment the very next day. I was ready! The lead hematologist at the University of Mississippi took lead. He greeted me and explained the treatment plan. He asked if I had questions or concerns and he answered each one. He made no promises but that his team would be available day and night to assist during my chemotherapy journey, and they were.

My first two chemotherapy treatments were good, but constipation or paralyzed gut was an issue. The team came up with a solution and allowed me to have the final say in the matter. Yes, I had a choice in the matter. The following two chemotherapy treatments were great. I was then introduced to the radiation team. My initial visit was scary because I did not know much about radiation and its effects. All I remember is the long-term effects. The lead radiation oncologist Dr. Vijay came in and made me laugh. He explained the step-by-step process of radiation, again. I was still scared. He said goodbye and told me that he would meet with me again after my final chemotherapy session. During the next visit, he explained my PET scan results and my body’s response to the chemotherapy. He seemed delighted about my results, but I was not. I had not heard of the total resolution of the mass. He explained that radiation would do the rest. Several days later, he called and explained that radiation was on hold. A debate was being held to discuss my case. I was amazed. The team set a date and the debate was held, chemotherapy versus non-radiation. In the end, I was given a choice. My decision was simple, to follow the doctor’s advice.

After my fourth chemotherapy treatment, I was scheduled to see the radiation team. I was still unhappy because I had not received the good news of a clear PET scan. I remember meeting the doctors and feeling terrified because I did not want to do more treatments and radiation. The doctors noticed my fearfulness and tried to relax me by reflecting on my courageous journey thus far. For the first time since my diagnosis, my husband and I were able to see in the picture the size of the bulky mass. I was amazed. In my mind, I pictured it much smaller. I stopped being fearful and became interested in what the doctor had to say. The doctors spoke in-depth about the treatment, my response to the treatment, and the next steps of my treatment. He explained the radiation process, possible side effects, and improvements with radiation over the years. He allowed us to ask questions and share concerns.

During the next visit with the radiation team, my last scan results were in. Again, I was disappointed because my scans did not show a total resolved mass. The doctors were encouraged and offered more detailed information about my radiation treatment. A team of doctors had recommended that I complete four weeks of focused radiation. It was explained that short-term side effects would be minimal. We viewed the scans to get a better view of the remaining activity in my body. I was finally ready to move to the next steps with radiation. During the next step, I was prepped and marked for my radiation treatments. I was checked for proper breathing techniques while undergoing radiation and directed to breathe out of my mouth and not my nose. This was the hardest task in my mind because I was overthinking the process. I was also introduced to other teammates who would help me during my radiation treatments. Everyone was very kind and knowledgeable about my diagnosis. Skin care education was also given during this visit.

I was awaiting my next appointment, but, instead, I received a conference call about a debate that was going to be held about my diagnosis, chemotherapy response, and future treatments. I was confused, but I was in awe. I felt as if I was in the care of specialists - research, debate, and all for me. The lead doctor told me that a fellow doctor had challenged his decision about radiation treatments. He stated that he would allow the other doctor to present research/rationale against radiation treatment, and he would present his research/rationale on the need for radiation. My husband and I were invited to the live debate. There would be doctors, nurses, and my support team. All of this is just for me. I felt so honored and blessed. This team of doctors was treating my case with research. My cancer journey had a purpose; purpose beyond me, a 51-year-old female with NHL.

Recommendations made after the debate

After reviewing all of the data, a consensus recommendation was made to repeat the PET-CT six weeks from the EOT PET-CT on 6/1/22 and approximately eight weeks from the actual EOT, which had already been ordered and scheduled for 7/13/22. If the PET-CT at that time showed the lesion to be increasing in size or FDG uptake, the team would then consider a biopsy to determine if the patient had refractory disease. If the lesion at that time was improving or unchanged, the team would continue to recommend active surveillance.

Follow-up with the patient and her husband

The PET-CT on 7/13/22 showed further continued favorable response to therapy with a residual low-level update in the index lesions as detailed. Left paratracheal conglomerate: SUV_max_ 1.4, 4.8 × 3.3 cm; previously SUV_max_ 2.0, 4.6 × 3.7 cm; left anterior mediastinal mass: SUV_max_ 1.5, 4.3 × 2.3 cm; previously SUV_max_ 1.5, 4.2 × 3.1 cm. Deauville score of 3. Ms.TCF was most recently in the radiation oncology clinic on 10/20/22 where she was doing extremely well with a Karnofsky performance status of 90-100, Eastern Cooperative Oncology Group performance status of 0, and both clinically and radiographically without evidence of disease. The plan is for continued active surveillance.

## Conclusions

This was a unique opportunity to offer our patient not only access to our clinical decision-making as colleagues but also an in-depth look at the way we care about her life and long-term survival. The MDT meeting with the incorporation of the patient allowed us as care providers to ultimately navigate the best treatment option for the patient, and in real-time allowed our patient to be part of that shared decision-making. Because of meetings like these, we as a team were allowed to improve our knowledge as new data emerge that supports different treatment modalities. Ultimately, due to the rates of CR and PFS with DA-EPOCH-R and the pros and cons discussion of radiation in this situation, it was felt that we could place her on active surveillance and forgo RT. In addition, it was useful to review the data that demonstrate a single EOT PET-CT has poor sensitivity. Her follow-up PET-CT months after the initial EOT PET-CT showed no further active disease.

It is to be recognized that the kind of shared decision-making described in this communication is very important in providing the best patient and family-focused cancer care. Such inclusiveness improves balancing the best scientific evidence with the best patient/family preferences and can lead to improved outcomes not only in survival and locoregional control but also in the quality of life and patient/family satisfaction. The example of the shared pathway described here can serve as a guide to our future improvements. Having the patient and her family present at the conference provided a different engagement with the team, which regardless of the patient’s HL, evokes the ability to have a shared and honest discussion regarding the best next step. For hematology and oncology patients, treatment pathways can at times be clearly outlined. When we as providers enter that gray zone in which pathways may be changing or unclear, we must come together as an MDT to present the most current literature that allows for the best care. Having the patient be a part of these discussions brings a certain trust to this shared decision-making. Taking what we have collectively learned, this will be a conference we look forward to continuing to offer when there is a need for this shared collaboration. This MTC ultimately benefited our patient, and, in doing so, we as providers benefitted and were strengthened as a care team.
